# Safety issues in post-discharge care of older patients in general practice: an ethnographic study

**DOI:** 10.3399/BJGP.2024.0284

**Published:** 2025-05-20

**Authors:** Rachel Ann Spencer, Zakia Shariff, Jeremy Dale, Graeme Currie

**Affiliations:** Warwick Applied Health, Warwick Medical School, University of Warwick, Coventry; Warwick Applied Health, Warwick Medical School, University of Warwick, Coventry; Warwick Applied Health, Warwick Medical School, University of Warwick, Coventry; Warwick Business School, University of Warwick, Coventry

**Keywords:** ethnography, Functional Resonance Analysis Method, general practice, patient discharge, patient safety, qualitative research

## Abstract

**Background:**

Post-inpatient discharge is a risky time for older patients, especially those with polypharmacy and multimorbidity. General practice care at this time, including the processes for managing hospital discharge summaries, lacks standardisation and is of variable quality. Understanding these processes will support the design of interventions and guidance to improve general practice management of the post-discharge period.

**Aim:**

To understand and visualise how ongoing care for older people after discharge from hospital is organised in general practice, including the processes for managing discharge summaries.

**Design and setting:**

Rapid ethnography study in 10 general practices in the West Midlands, England.

**Method:**

We mapped the practices’ systems of post-discharge care. Data sources included informal conversations with staff, practice policies, and direct observations of discharge summary handling. Fieldnotes and quotes were subject to an interpretivist framework analysis. A systems modelling technique (the Functional Resonance Analysis Method) was used to present visual representations of the professional roles working in these complex systems.

**Results:**

Three basic typographies of system emerged based on professional roles: GP-led, pharmacist-led, and administrative-led. We report on three themes that weave around the Functional Resonance Analysis Method process maps: comfort with demands of administrative role; general practice team dynamics; and interaction with patients.

**Conclusion:**

General practice systems for inpatient discharge summary processing are complex and varied. New roles in general practices are being used extensively, often requiring significant input in supervision by GPs. Our findings highlight safety features of different systems and should help practices understand the advantages and limitations of models they work within.

## Introduction

Continuity of care for older patients who have had recent hospital inpatient care is a core function of general practice. Yet the handling of clinically appropriate requests carried in discharge summary letters is variable, resulting in a potentially unsafe transition of care from acute hospital admission back to routine general practice.^1^ For those aged ≥75 years an 8% rate of harm has been identified.^2^ While most harms are mild (such as a missed opportunity to treat symptomatic conditions such as anaemia) or moderate (for example, failure to stop medicines that caused subsequent kidney injury or further admissions), discharge-related harms in primary care do include death.^3^ As populations age,^4^ increasing numbers of discharge summaries for an increasingly complex cohort need to be safely processed in general practice. In 2020/2021 in England there were 6.2 million finished admission episodes for patients aged ≥65 years (39% of total finished admission episodes), of which 2.6 million (42% of the total) were emergency admissions.^5^ Extrapolating from these data, an average general practice of 10 000 patients^6^ will receive 1092 discharge summaries each year for people aged ≥65 years, equating to around four discharge summaries each working day.

In England, national standards for discharge summary content exist,^7^,^8^ but there are no agreed standards of care or interventions for primary care management after discharge and no routine follow-up appointment scheduling. GPs lack mandate or structure to allow them to devote time to this activity. Despite, or perhaps because of, the speed of discharge summary processing in primary care (approximately one working day), omissions occur.^2^

General practices often use a multidisciplinary team approach to older people’s health care,^9^ particularly for hospital admission and discharge, but evidence of effectiveness is mixed. The roles and relationships in the UK general practice team are in a state of flux.^10^ Administrative staff have been encouraged to take on greater responsibility in relation to documentation handling^11^ and in some instances this had led to them taking an active role in post-discharge care. In England, the Additional Roles Reimbursement Scheme (ARRS)^10^ has recently rapidly expanded the types of roles in general practice and has increased system complexity substantially.

**How this fits in**

General practice systems for post-inpatient discharge care of older patients are poorly understood. We address this with an ethnography and systems analysis of 10 general practices. Some core functions were common across all practices, but in functions relating to staff role there was a high degree of variability; we illustrate this with three models. Discharge summary management is complicated, and transfer of workload does not always work well or in the way it might for other paperwork in general practice. This study highlights potential safety concerns and paves the way for intervention development targeted at helping practices to set up and monitor their own systems for managing older patients following inpatient care.

There are many plausible routes to reducing errors and harms in post-discharge management, including design of safer systems; protected time and workspace; and better integration between primary and secondary care.^12^ In order to inform such work, we aimed to develop a full understanding of how inpatient discharge summaries are processed within a range of general practices. We used an organisational science approach to understand how human factors influence the safety of the systems observed. Our specific study objectives were to gather ethnographic data from a range of sources to characterise the processes; develop systems typographies and identify associated safety issues; and understand what interventions might be needed in general practice to improve safety.

## Method

Two researchers (an academic GP and an academic pharmacist) collected data from 10 general practice sites in Coventry and Warwickshire during summer 2022, purposively sampled for variation in practice size, geography (rural or urban), ethnic diversity, and socioeconomic status. Following rapid ethnography methods,^13^ data collection centred on the journey of the discharge summary around the general practice, including the points of outreach to patients and/or carers and other agencies.

Initial data collection included gaining a basic frame for each practice system, including who the key staff members were to talk to and any written protocols that the practice already had in place. These initial sessions told us about work ‘as imagined’.^14^

The two clinician researchers then visited each site for around a day to observe work ‘as done’,^15^ and conduct informal conversations with four groups of staff: practice managers and administrative team managers, administrative team members handling electronic copies of discharge summaries, GPs, and pharmacists and pharmacy technicians.

At the practices where we conducted the ethnography, nurses and nurse practitioners were not involved in managing discharge summaries.

Practice staff were watched as they worked with discharge summaries (usually in Docman, an electronic document management system), and ‘think aloud’ methods,^16^ along with a topic guide (see Supplementary Appendix S1 for details), were used to capture fieldnote data and quotes relevant to structure, processes, and outcomes in post-discharge care. Audio-recording was generally avoided to increase the candour of informal conversations, particularly with administrative team members. As part of an allied research strand,^17^ we also conducted searches for recently discharged older patients at the same practices. These searches sometimes highlighted systems problems with discharge summary handling — findings that were fed back into the ethnography.

Transcriptions of field notes including detailed quotes were subject to framework analysis using NVivo (version 12).^18^ One researcher first became familiar with the data, coding semantically. A coding framework (see Supplementary Box S1 for details) was developed with the wider project team, which included a business systems expert, a qualitative methodology professor, and a GP professor. Dual coding for quality control was undertaken by two researchers for 20% of transcripts. Final themes were discussed and agreed with the wider study team.

**Figure 1:**
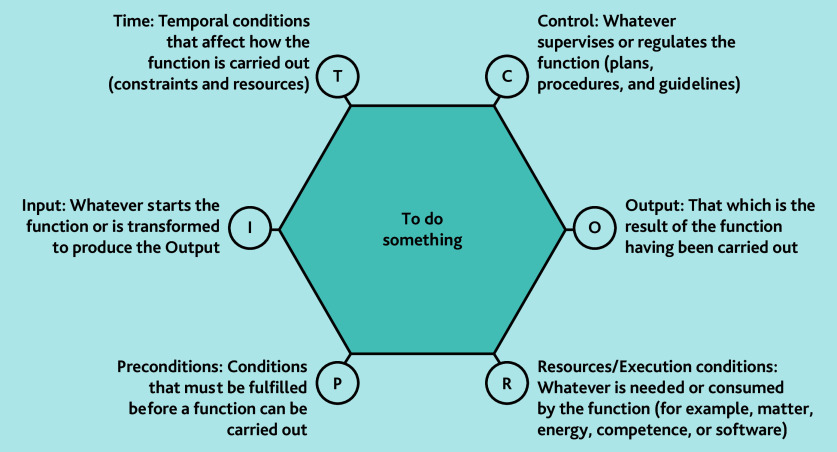
Core aspects of a ‘function’ as described in FRAM. Functions can be considered as points of action on a timeline of a process, for example, ‘receive discharge summary’, ‘send discharge summary to pharmacist’, and so on. Figure adapted from the *FRAM and Manual.*^20^ FRAM = Functional Resonance Analysis Method.

We used the Functional Resonance Analysis Method (FRAM)^19^ to create process maps with the FRAM Model Visualiser (version 2.1.6), which complements our framework analysis. Supplementary Appendix S1 details how FRAM was considered in the topic guide. FRAM, while still fairly novel in medicine, is often used in business systems analysis. It facilitates core functions of a system being explored in relation to their variance and inter-connection to each other, and from this a web-like diagram is created. In Figure 1, the centre of the hexagon represents the ’function’ (what is being done) and the lettered ‘aspects’ at its corners represent the factors that affect the function.

Codes relating to FRAM ‘aspects’ (control [C], resources [R], and time [T]) were applied to the fieldnote data in NVivo (version 12). Common ‘functions’ ascribed to hexagons were determined from the researchers’ familiarisation with the dataset (a similar process to the identification of qualitative themes). Bespoke FRAM diagrams were created for each practice by extracting the ‘aspect’ codes for each practice site. Differences and similarities between FRAM diagrams were considered across the set of practices and characterised into a typography.

## Results

The practices were typical of those in England, showing a variety of demographics and access to specific roles and resources that are relevant to post-discharge care (see Supplementary Table S1 for a summary of practices and informal conversations). Data collection involved 450 minutes in site initiation visits with senior practice staff and 1140 minutes in direct conversation (215 minutes with GPs [*n* = 9], 180 minutes with pharmacists and pharmacy technicians [*n* = 4], and the remainder with managerial and administrative staff [*n* = 13]). The 26 informal conversation contributors were of a variety of self-declared ethnicities: most were White British (*n* = 16) and eight were British Indian, with an average age of 43 years.

We present results of the thematic analysis of observation notes in the context of the FRAM models we created. Three key themes were identified (see Supplementary Box S1 for details of the coding framework). The ‘Comfort with demands of administrative role’ theme encompasses the following sub-themes: the enhanced role of administrative assistants, their training, their interaction with protocols, and how they work in teams. The ‘General practice team dynamics’ theme includes material on power and control, trust, new staff roles, and duplication of work. The ‘Interaction with patients’ theme includes access facilitators and barriers, continuity of care, enhanced access, and health literacy. Other themes (IT, workload, environment, and interactions with secondary care including inappropriate transfer of workload) did not differ substantially between practices and are not considered in this article. The first two themes implicitly relate to occupational dynamics, while the third theme was also found to be delineated by staff role, with practices and expectations for contact offered, differing by staff role. Therefore, we consider the FRAM models from an ‘occupational roles’ perspective. Initial bespoke FRAM models created for each practice revealed that, without staff role delineation ‘functions’ at the centre of each hexagon, there was little difference between the aspects of the functions between practices.

There were three broad system types (Box 1), each showing devolved levels of control based on lead staff roles. The remaining practices (*n* = 4) straddled two model types and/or were in a state of flux (see Supplementary Table S1 for details).

Box 1 **Simple summary of FRAM models**

**Table:** 

**Model A: GP-led (practice codes 17 and 18)**
GPs complete all key actions such as coding and medicines reconciliation but may delegate patient contact to administrators
**Model B: Pharmacist-led (practice codes 10 and 19)**
Pharmacists complete all key actions such as coding and medicines reconciliation but may delegate patient contact to administrators
**Model C: Administrative-led (practice codes 14 and 15)**
Administrators complete all key actions such as coding, medicines reconciliation, and patient contact

FRAM = Functional Resonance Analysis Method.

**Figure 2:**
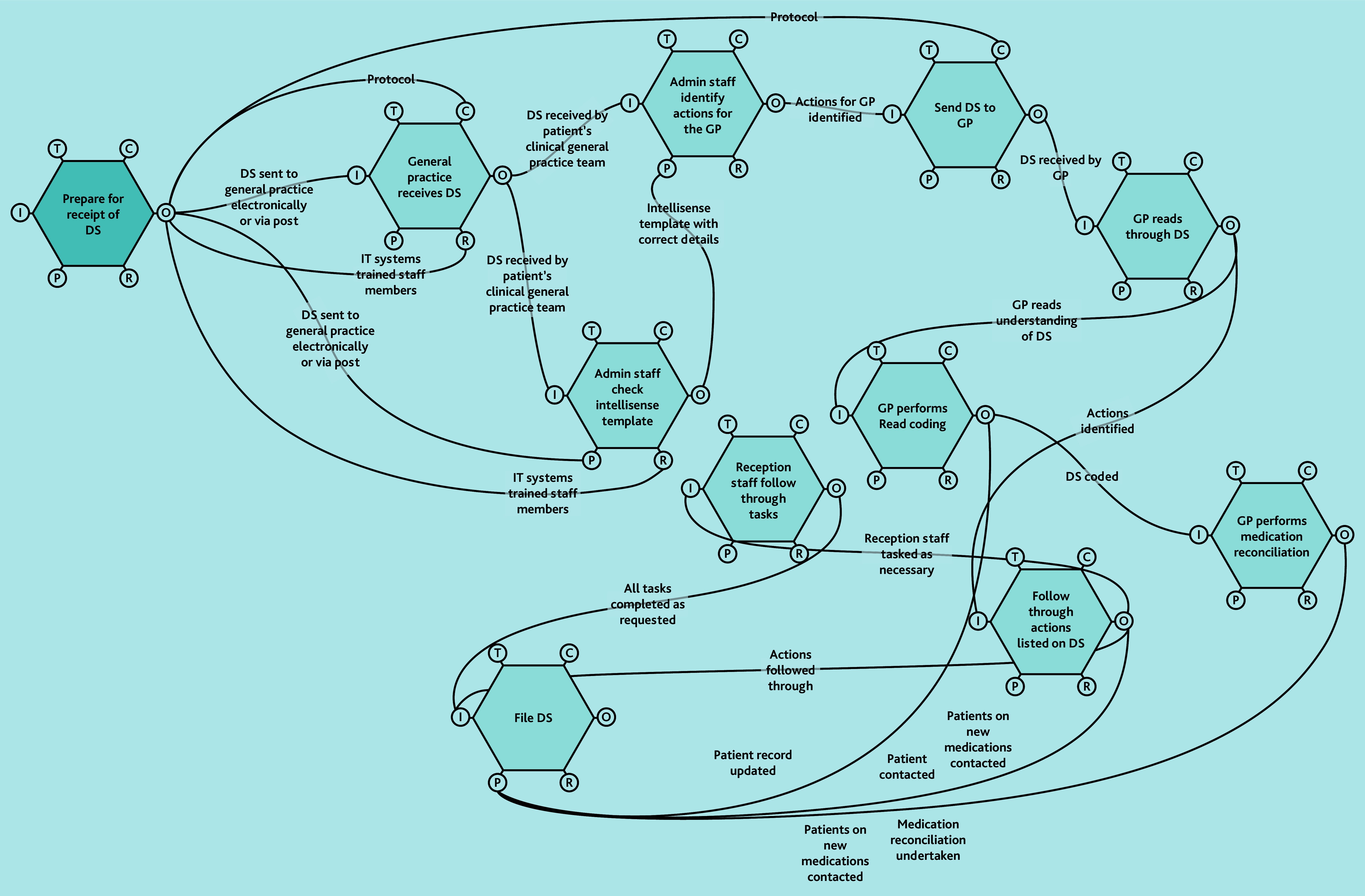
Simplified FRAM model of a type A GP-led system. Circle = aspect; aspects clockwise around the hexagon: T = time, C = control, O = output, R = resource, P = pre-condition, and I = input (see Supplementary Box S2 for details). DS = discharge summary. FRAM = Functional Resonance Analysis Method. Hexagon = function.

**Figure 3:**
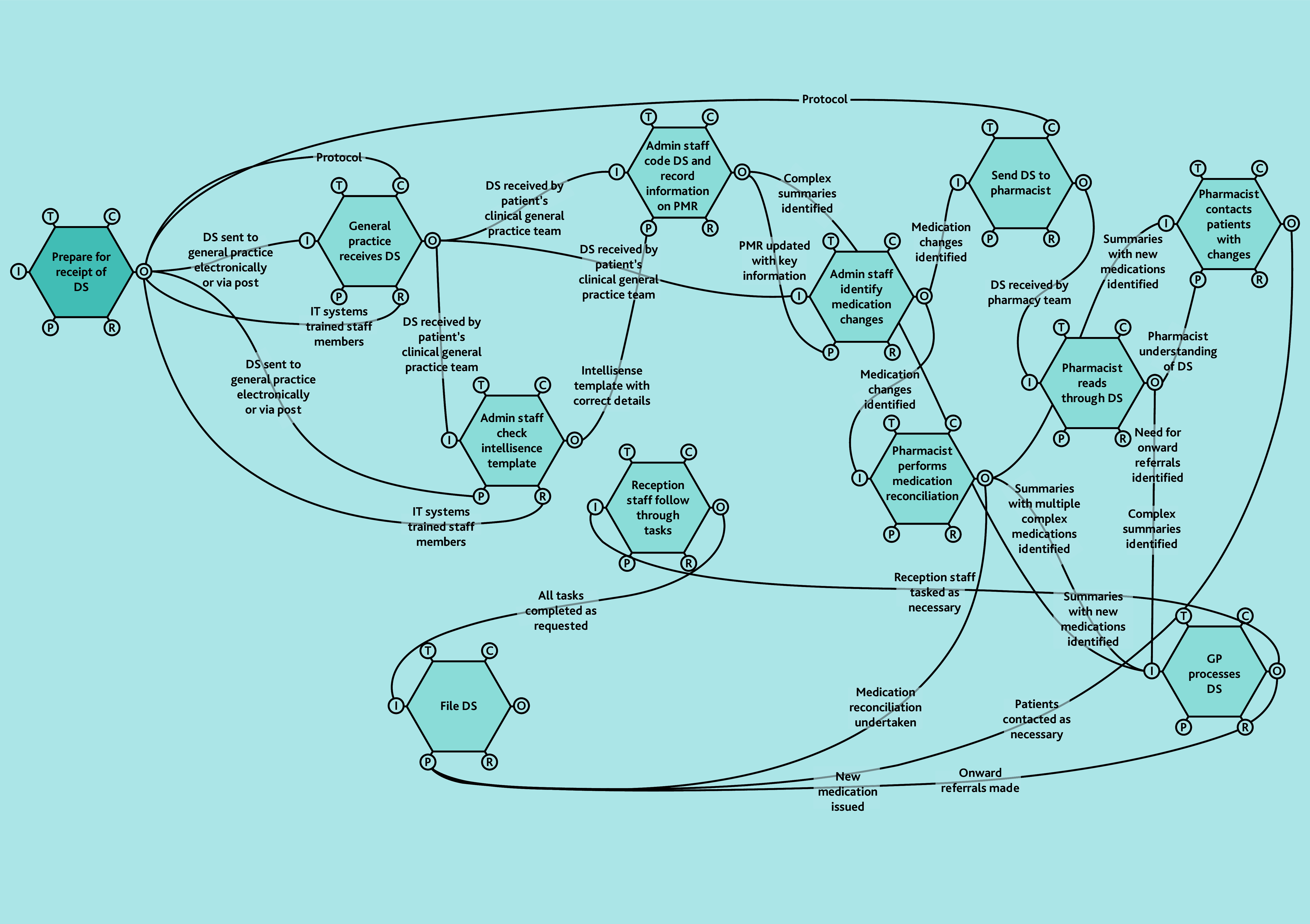
Simplified FRAM model of a type B pharmacist-led system. Circle = aspect; aspects clockwise around the hexagon: T = time, C = control, O = output, R = resource, P = pre-condition, and I = input (see Supplementary Box S3 for details). DS = discharge summary. FRAM = Functional Resonance Analysis Method. Hexagon = function. PMR = patient’s medical record.

**Figure 4:**
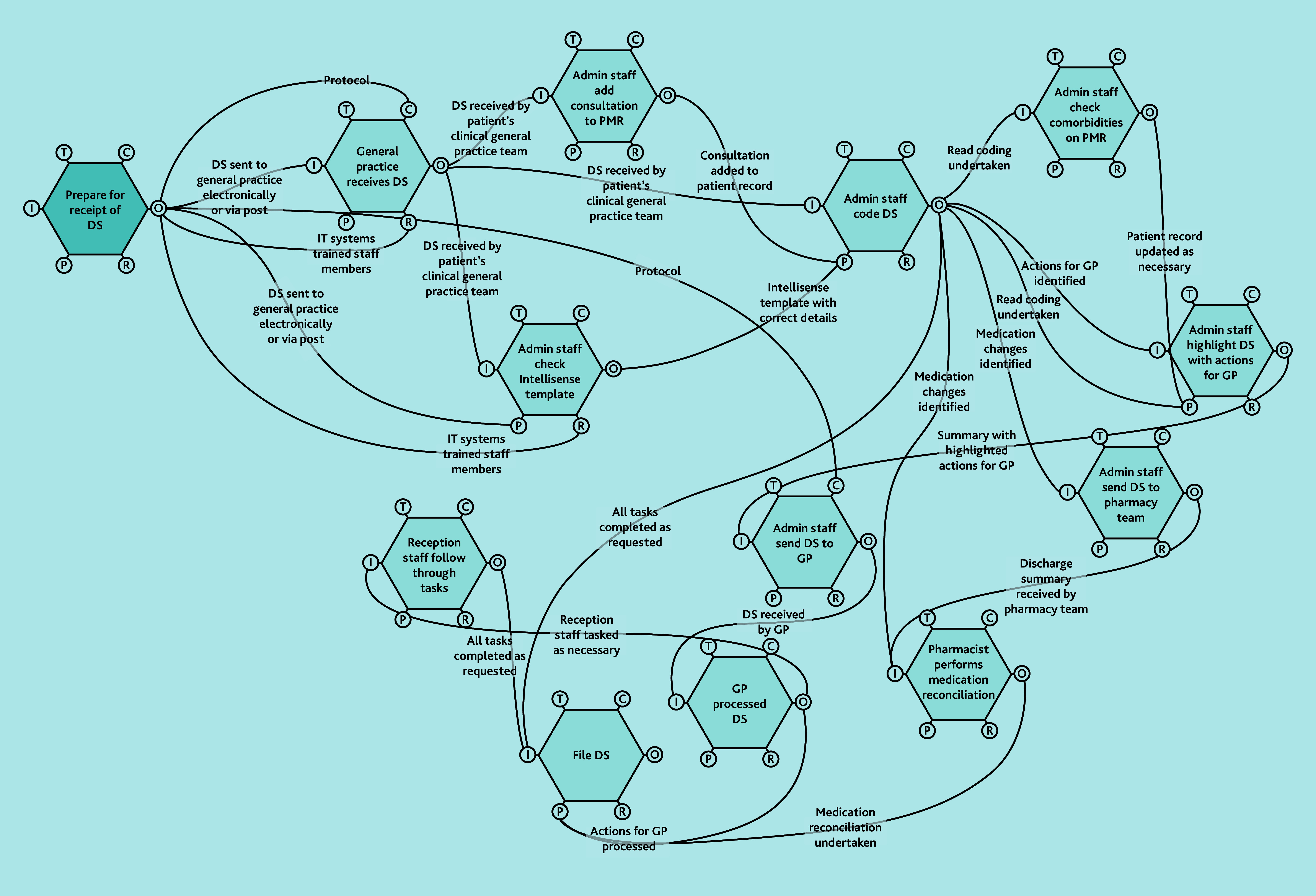
Simplified FRAM model of a type C administrative-led system. Circle = aspect; aspects clockwise around the hexagon: T = time, C = control, O = output, R = resource, P = pre-condition, and I = input (see Supplementary Box S4 for details). DS = discharge summary. FRAM = Functional Resonance Analysis Method. Hexagon = function. PMR = patient’s medical record.

A does not imply superiority over B or C. FRAM models (Figures 2–4) for each of these are described below. In all three types, the practice receives discharge summaries and administrative staff scan paper copies and remove duplicates. The models begin to differ when administrative staff either process the summary or forward it to a clinician. In type C models, administrative staff process the summary, whereas in types A and B, summaries are automatically sent to clinicians (a GP or pharmacist, respectively). The model types were not mutually exclusive; all possible combinations of models were represented among the practices studied. Only pure type A (GP-led) systems were associated with practice size (small). Some practices were described as being in a state of change (especially during staff turnover). Each model is outlined in Figures 2–4 (see Supplementary Boxes S2–S4 for a detailed description of functions and their aspects, which may be useful for those unfamiliar with general practice organisation in a UK context).

Below we draw on the qualitative findings to provide further insight into how staff are working together within each model type. See Box 2 for key themes and subthemes. Quotes are followed by staff role (A = administrator; GP; P = pharmacist; and PT = pharmacy technician) and practice code; where numbers are provided after staff role (for example, A1), this indicates that ≥1 individual of this staff role was present during interview.

Box 2 **Themes and subthemes reported in the Resultsa**

**Table:** 

Theme	Subthemes
**Comfort with demands of administrative role**	Enhanced role of admin (including training) Protocols Role of colleagues in provision of support of knowledge
**General practice team dynamics**	Double checking/duplication Pastoral support from colleagues Power and control Roles (additional in general practice) Role sharing (interpersonal relationship) Trust
**Interaction with patients**	Access/enhanced access Continuity of care Patients’ health literacy

a For a full description of the coding framework see Supplementary Box S1.

### Type A: GP-led FRAM model

In type A models (Figure 2), discharge summaries are only handled clinically by GPs (see function hexagons in the upper-right part of the diagram). Practices using a type A model either did so because ARRS staff were not available to them or because they felt that this was the model with the highest degree of patient safety. Their general practice team dynamics were both affected and caused by the choice of model. One practice had reverted to this system, tightening control by the ‘top of the tree’ because of previous administrative errors. In these systems, leaders are wary of those with insufficient clinical acumen being asked to assume responsibility:

*‘It doesn’t need to be a GP who sees them after discharge but off the back of that appointment come a load of questions that mean it needs to be a GP conversation.’* (GP11)

*‘The way they do things now works well — very little gets missed because of the way we do things.’* (GP17)

Power structures have a strong input into process design, and often the power is controlled by GPs:

*‘He* [lead GP] *tells us it’s his own design and that the priority is “just to make sure nothing gets missed”.’* (GP18)

Administrative staff are likely to refer up a hierarchy:

*‘If I’m ever even half a per cent unsure then it goes to a doctor … fear of missing something otherwise … All discharge summaries go to doctors, I don’t even bother to read them, I just send them to the script doctor.’* (A1 13)

How GPs in type A systems complete workflow varies. Depending on the team dynamics, they often balance transfer of work burden to their administrative staff against their lack of dedicated slots:

*‘Try not to send too many tasks, just cut out the middleman and do it myself … when there are big changes, I just call them and there is no appointment booking.’* (GP18)

Text messaging (SMS) was a common form of direct communication from GPs to patients to save time:

*‘The good ones* [clinicians] *send an AccuRx* [SMS] *to the patient directly.’* (A1 11)

Interactions with patients in type A systems are often built around longitudinal continuity of care, with workflow based on ‘nominated/named’ GP or sometimes ‘episodic continuity’ of whoever saw the patient last. How practices define the appropriate GP to receive the discharge summary varies (Box 3). However, it is considered especially important to get this right for follow-up of new major diagnoses:

*‘We try to send to the regular or registered GP … anything serious like that* [a cancer diagnosis] *we send to the GP, especially if they know them.’* (A1 11)

Box 3 **Example of variation in function ‘send summary to the GP’ between practices**

**Table:** 

**‘Appropriate GP’** definition varies across practices: Nominated GP (from banner in patient’s electronic records) GP who saw the patient last GP who requested the referral to hospital GP who is named on a written protocol
**When** the summary is sent to the GP varies across practices: Send before SNOMED coding Send after SNOMED coding Send only if there are tasks that cannot be performed by anyone else Send after the pharmacist has made medication changes

Sometimes continuity is sacrificed for timeliness. GPs in a practice later re-distribute summaries among themselves to improve continuity by making colleagues aware of admissions.

### Type B: Pharmacist-led model

In type B models (Figure 3), discharge summaries are handled clinically by pharmacists (see function hexagons in the upper-right part of the diagram). All practices using pharmacists have channels of communication between pharmacists and GPs (see lower-right hexagon). In some observations we witnessed single discharge summaries passing through the hands of as many as four staff members before they reached the top of the tree (usually a GP). Pharmacist roles have specific value for medicines reconciliation:

*‘I’m not blowing my own trumpet, but there’s things that we look out for that others don’t.’* (P19)

Pharmacists were observed undertaking different activities from GPs during medicines reconciliation (such as writing summaries of medicines changes in the facing record and concurrent medicines optimisation). We observed linking medication with problems, using STOPP and START criteria,^21^ and checking the safety of drugs added by the hospital. Pharmacist-led models affect the general practice team dynamics; some GPs still felt responsible for overseeing the decision making and actions of others when they had been passed a document (leading to duplication):

*‘It comes highlighted but I read through it anyway just to double check what’s happened … they’ve put an entry in the notes with the changes then I’ll go on the discharge summary and EMIS and check — it’s time consuming and tedious.’* (GP12)

Conversely, some felt better able to trust clinical colleagues’ decisions and actions:

*‘I don’t usually double check the medications — if the pharmacist says they’ve changed the medications, I’ll trust them.’* (GP19)

Administrative staff often tried to save time for clinicians working in complex mixed models by, somewhat laboriously, typing free-text instructions:

‘she puts a note for [pharmacist] indicating what needs to be done on the summary and also writes down the name of the GP that she needs to forward on the summary to *“please update meds as per instructions for GP, also see pharmacy comments. Then please forward to* [pharmacist initials]*.”’* (A19; Roman indicates observational note)

Many discharge summaries still ended up with a GP, especially where medical complexity was involved:

*‘I’d say ninety-nine per cent of the older people ones get to a GP* [because of medication changes and actions]*.’* (A12)

ARRS pharmacist roles are relatively new; there are power conflicts in what they are ‘allowed’ to do in relation to discharge summaries:

*‘It’s not a medicines review, I can’t do those, so I don’t code it as that.’* (PT12)

*‘The pharmacist only adds new medications on as a “single repeat”. This acts like a “safety net” so that it is bought to the attention of the GP at the next prescription.’* (GP10)

*‘He’s not allowed to do onward referrals (to physios, et cetera) … He has to speak to a GP for this, simple blood tests he can do.’* (P16)

Pharmacists train in ‘buddying’ models with GPs and disseminate their training to PTs despite specific primary care network training pathways. Team stability and pastoral support are important:

‘He works from the top and his colleague [name] works from the bottom (they usually work alongside each other) … they can bounce ideas off each other because they are in the same room.’ (P13; observation)

Communal electronic inboxes allowed for team working, which suited levels of experience:

*‘There’s no pressure’* everybody can see other’s tasks on Docman *‘the pharmacist went and cleared my inbox while I was away.’* (PT12; Roman indicates observational note)

Pharmacists sometimes used their booked slots to interact with patients, but did not call patients routinely. Where direct contact failed, they offered continuity via SMS:

*‘Texts make everything easier — someone called* [name] *offered help — it gives them someone to come back to.’* (P13)

### Type C: Administrative-led FRAM model

In type C models (Figure 4), discharge summaries are screened by administrative staff (see function hexagons in the upper-right part of the diagram) before deciding what input, if any, is required by clinical staff (see lower and central hexagons and Box 3). All practices using type C models had mechanisms for administrators to ask for help from clinicians but discharge summaries without significant diagnoses or actions could potentially be filed by administrators. We collected data related to long experience and self-expressed lack of training need under a theme named ‘Comfort with demands of administrative role’. In type C models, administrators described comfort with the demands of their enhanced role including managing test requests, referrals, and direct contact with patients:

*‘Knowledge develops in that time.’* (A14, 15 years’ experience in post)

There was still the option to workflow to a GP:

*‘Our aim is to send as little as possible to the GP … at most it’s three a week that are sent to GPs.’* (A1 15)

This workload complexity has trade-off:

*‘Sometimes it’s overwhelming, especially if it’s a difficult or long one, sometimes I put those ones off until tomorrow so I can look at it with a fresh head.’* (A1 15)

And administrators can strain against the limits of their role:

*‘I’m trying to cut it down, if it’s a complex letter I find it hard to cut it down. Hard to know what to put in and what I shouldn’t.’* (A10)

Backlogs in filing in Docman were evidenced in all system types, including type C practices:

*‘There are more than one thousand in the inbox right now and they have a backlog of about three weeks.’* (A1 15)

Type C models often demonstrated strong, longstanding, symbiotic relationships between GPs and senior administrators. The 12 administrators had worked for a mean of 10.5 years each in health care and were confident in their work because of experience. Most senior administrative staff had undergone training for their enhanced role externally; sometimes a delay to training meant staff started on the job. Senior administrators trained apprenticeship style with a GP buddy, particularly at smaller practices:

*‘*[Administrator] *is dependable — she knows and follows the process. At the beginning we worked together for a few months — she kept a list of ones she was unsure about.’* (GP12)

Senior administrators used external training to train other, more junior staff:

*‘I started off watching people doing it, then I did easy stuff to start with like orthopaedics, ENT* [ear, nose, and throat], *dermatology, eye. Every letter initially was checked by somebody.’* (A16)

Traditional hierarchies are evolving into a flatter power structure, with recognition of responsibility, status, and changes in role titles:

*‘Our reception staff are all “care navigators” now, they’re incredibly experienced.’* (GP15)

Transitioning of professional knowledge and expertise is complex and not always complete:

‘He indicated with non-verbal dismissive gesture … *‘I wouldn’t highlight any of that … I would code a diagnosis.’* (GP14; Roman indicates observational note)

## Discussion

### Summary

We used a novel combination of methods to develop insights into how discharge summaries for older patients after inpatient care are managed in general practice. Although recognised as being a priority workstrand, the receipt of such documents did not necessarily trigger a timely clinical assessment of their content and follow-up. Indeed, filing backlogs of several weeks were not uncommonly observed and this is an unaddressed patient safety issue. We encountered the long-favoured patient safety technique of double checking by administrators^22^ and those in clinical roles. Patient safety is the driving force behind duplication, but inefficient use of scarce workforce time must be considered, in itself a patient safety issue. Trust and patient safety are closely interwoven within the choices involved in the set-up of these duplicative systems^23^ influenced by power dynamics in practices. We found the power balance between staff roles to be in flux, with practices migrating between organisational process model types A (GP-led), B (pharmacist-led), and C (administrative-led). A summary of key patient safety issues associated with these model types is presented in Box 4. In model C there is delegation (in established dyadic models) and in model B there is substitution (of GPs with pharmacists), which requires control and regulation to be safe.^24^

Box 4 **Simple summary of theme-linked strengths and weaknesses of model types**

**Table:** 

Model type	‘General practice team dynamics’	‘Interaction with patients’	‘Demands of the administrative role’
A: GP-led STATUS QUO	Safe, controlled, and onerous on GPs	Higher levels of continuity possible Access may be more difficult	Higher comfort level Possibly lower job satisfaction
B: Pharmacist-led SUBSTITUTION	Specific medicines expertise Potential safety risk (lack essential knowledge) Supervision issues Potential duplication	Access may be easier (booked slots) Continuity possible	Higher comfort level Possibly lower job satisfaction
C: Administrative-led DELEGATION	Relieves clinicians Potential safety risk (lack essential knowledge) Least control Training required	Access may be quicker Continuity may fail Access to clinician less likely	Potential higher job satisfaction Lower comfort level

The FRAM methodology enabled characterisation of the three distinct models and the processes and roles within them. General practice staff and researchers wishing to study or change models should review the aspects of the functions in the section they wish to modify or observe for potential knock-on effects on patient safety. Some practices changed between model types during the study, reflecting the extent to which practices’ systems of working are changing in the context of workforce and workload pressures.^25^

### Strengths and limitations

A strength of our work is the large number of sites and privileged access to observe general practice back-office systems. We were able to gain candid views in one-to-one settings with clinicians and administrators. Observing consultations in general practice post-discharge was not possible because such consultations happen reactively and *ad hoc*, and some of the consultations happen in out-of-hours care because of their emergency nature. We instead used recall by the clinical informal participants and ‘think aloud’ methods while they were at their usual working desks with the clinical information systems open. As an academic pharmacist and GP team, we were able to bring our experience to bear to rapidly understand practice systems through a FRAM lens. All the sites were in one geographical area, but this did not diminish the variety in systems observed. Our method did not aim for saturation on the issues associated with role-based models and there is scope for further study of these systems from a human factors perspective. Despite separate coding processes, our qualitative themes and subthemes had some natural overlap with the ‘aspects’ of functions in the FRAM diagrams and allowed clarification and understanding of what pressures are placed on the ‘aspects’ and how they interlink. To our knowledge, FRAM has not previously been used in a general practice context (although it has been previously used to describe discharge care from a hospital perspective).^26^–^29^ We have made a substantial contribution to the sparse literature surrounding understanding of GP document handling and identified its complexity and the potential scope for error.

### Comparison with existing literature

To our knowledge, this is the first study to clearly set out a typography delineating how general practices manage discharge summaries for older people. This has importance in terms of understanding how different models can affect the safety and quality of care that is subsequently provided to such patients. Type A models may help to ensure episodic continuity^30^ if they are coupled with rigorous administrator work-flowing of summaries to the ‘correct’ GP (Box 3). There is a trade-off between expertise and time pressure on GPs and potential delay in processing.

Type B models have the potential to tighten prescribing safety because pharmacists are medication reconciliation specialists.^31^ They reduce pressure on GPs yet increase capacity for contact with patients where there are dedicated diarised pharmacist appointment slots. Workload may be duplicated, depending on level of trust in pharmacist actions and decisions.^32^ Current constraints with pharmacist employment in English practices mean that they work across multiple sites, role-share, and provide disjointed cover across the week.^33^ Inequity in pharmacist provision means some practices lack access, reflecting a national shortage of suitably experienced pharmacists (partly addressed with the employment of non-clinically qualified pharmacy technicians).^34^ Jurisdiction in supervision models of pharmacists and pharmacy technicians^35^ working in general practice is not fully agreed; primary care leaders need to take care that they are not given ‘scut’ work.^36^

Type C models may reduce pressure on clinicians and encourage career development for administrative staff. Five per cent of receptionists reported dealing with discharge summaries in a 2017 national survey,^37^ but this percentage may have increased since then. Administrative staff feel under pressure with highly complex cases despite thorough training both internally and externally. There is the potential for a backlog of discharge summary processing when key administrative staff take annual or sick leave. Time pressure also intensifies when administrative staff are balancing several roles.^38^ Most practices in our sample employed at least the equivalent of a full-time administrator to handle discharge summary processing; larger practices were better able to support administrative assistants entirely dedicated to back-office functions.^39^

### Implications for research and practice

Changes to discharge summary processing could be described as ‘task shifting’,^40^ a concept that has been extensively applied in UK general practice and studied between clinical roles (especially GP to nurse)^41^ but is less well understood for pharmacists,^40^ administrative assistants, and receptionists.^42^ Although often driven by the need to make cost savings and lack of GP capacity, there is an opportunity cost of loss of jurisdiction and GP time used in training and oversight. Efficiency gain must be balanced with a patient-centred approach, which ensures continuity and clinical safety, and links the processing of discharge documents to more tacit knowledge that clinicians have about patients (a concept akin to mindlines).^43^

**Figure 5:**
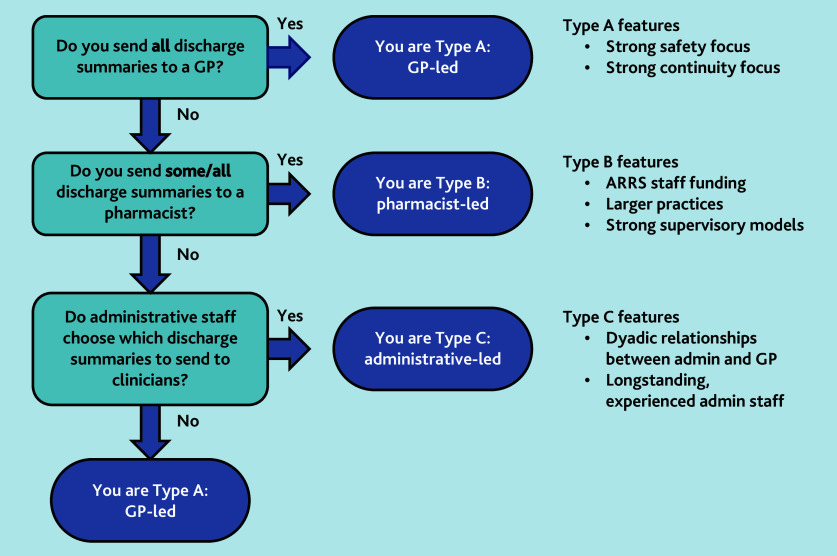
Understanding a practice’s model type for managing the discharge summaries of older patients. ARRS = Additional Roles Reimbursement Scheme.

Practices could use Figure 5 as a starting point to classify their approach to managing inpatient discharge documentation for older patients and to consider protocolising their systems, reviewing, and, if necessary, updating the training of relevant staff. This is especially important when change is being planned, staff mix changes, or if significant events occur. This work is part of the wider GP-MATE intervention co-design study (National Institute for Health and Care Research reference: 301328).^17^ We will carefully consider how our GP-MATE intervention fits within the role-based systems we have identified and will develop bespoke implementation packages for practices taking part in our pilot study.

We met our study aim to understand how practices organise their post-discharge care via an organisational science approach. We found role-based issues to be of greatest interest in terms of systems variability and hence of greatest potential to affect patient safety. A priority for future research, therefore, is the development of post-discharge interventions that can be integrated into the three system models described and that tackle outcomes of high relevance to patient safety in general practice.

## Supplementary Information


