# Supplementary material for: Safety issues in post-discharge care of older patients in general practice: an ethnographic study
Source: Br J Gen Pract. 2025 May 20;75(755):e412–22. doi: 10.3399/BJGP.2024.0284 (PMC12117506; doi:10.3399/BJGP.2024.0284)
Supplement: Supplementary file 1 [file BJGP.2024.0284_suppl.pdf]

Supplementary Materials

Appendix 1 (S1) - Verbal Consent Script – Informal Conversations with practice clinical staff to develop process maps

Before we talk further, I'd like to make sure you're happy to take part in this study. As you know, I'm conducting conversations in the practice as part of the GP-MATE study. To recap, the study is looking at how older patients interact with their general practice after discharge from hospital. It has been approved by the Essex ethics committee.

If you agree to take part, I'd like to ask you some questions (or continue talking with you) about how post-discharge care of older people is organised in your practice. This conversation will be used as part of my data collection for the study, and may be included publications and reports.

Would you be happy to continue talking? [Await confirmation]

If so, would you be happy for me to write notes about our conversation? [Await confirmation]

I will not write down your name or any identifying details. I will keep my field notebook secure and no one outside the study team will have access to it.

Do I have your permission to write down direct quotations of what you say? Do I have your permission to write down paraphrased quotations of what you say? It's okay if you don't agree to be quoted. [Await confirmation]

Before we continue, I just want to emphasize that you don't have to answer any question you'd prefer not to respond to, and that you're free to end the conversation at any time. Also, if you'd like to withdraw your consent to participate at any point during the conversation, or within the next week, that's fine too – just make sure to let me know. I'll leave you with my contact details.

Are you happy with all of this? [Await confirmation]

Do you have any questions for me? [Await questions]

Alright, let's continue.

1) **We can start when a patient is first discharged from hospital. Can you talk me through the process of post-discharge care at the practice?**
   a. How do you find out that a patient has been discharged?
   b. Do discharged patients come to your attention any other ways than via Docman? ("we will discuss Docman in a bit")
   c. Do you ever see/phone patients after discharge? (if so what factors would prompt you/them to contact)
   d. Are there any special procedures for older patients or other vulnerable groups?
   e. Are there any outside agencies that are also seeing the older/vulnerable groups?

2) **Do you know anything about how the discharge summaries are processed by the admin team?**
   a. How do the summaries get to you?

   b. Do you know who in the admin team are responsible for processing the summary/how they do it?
   c. Are there any on-call arrangements for urgent/specific documents?
   d. Do admin do any coding/medication changes?

3) **How do you approach the discharge summary?**
   a. Are there any specific area you look for when processing the summary/ order in which you approach it?
   b. Which staff role do you think needs to deal with discharge summaries?
   c. How long does it usually take to process discharge summaries?
   d. Do you undertake any coding? If so, how long does this take and have you received any training for this?
   e. Does anybody else check-up on the work you have done?
   f. When do you contact patients about discharge summaries?
   g. Do you ever see patients – who initiates the consultation?

4) **How are medication changes usually dealt with?**
   a. How do you make medication changes?
   b. What resources do you usually refer to? How long does this task usually take?
   c. Are patient/carers involved in changing medications?
   d. Are any other healthcare professionals involved in this task?

5) **How are requests for follow up/tests/investigations handled?**
   a. How is this task usually approached?
   b. Is anybody else in the team involved?
   c. Who is responsible for following up tests/investigations?
   d. Who is responsible for any communication regarding tests/investigations to the patient?

6) **Are there any other tasks which need to be completed before a discharge summary is filed?**

The following follow up questions were taken from the FRAM handbook and used as a template for follow up questions when discussing each activity described by the practice team:

1. When do you start this activity? What "signals" that you can begin?
2. How do you adjust the activity to different conditions? How do you determine how and when to adjust?
3. How do you respond if something unexpected happens? For example, an interruption, a pause required by a more urgent task that takes priority, a missing resource, etc.
4. How stable is staffing? Is staff allocation permanently assigned or adjusted daily? What happens if staffing is short?
5. How stable is the environment? Supplies? Resources? Demands? Etc
6. Are there often undesirable conditions that you have to tolerate or get used to?
7. What preconditions are usually met?
8. Are there factors that are taken for granted?
9. How do you prepare for your work (documents, instructions, colleagues, etc)? What do you do if these resources are not available?
10. What information do you need (equipment, services, etc)? What do you do if this is not available?

11. How does time pressure affect your work?
12. What skills do you need? Does everyone performing this work have these skills?
13. What is the optimal way to perform this work? Is there an optimal way?
14. How often do you change or adjust your work?

Supplementary Box 1 (S1) – Coding Framework

Bold = theme Plain text = subtheme (can code under this is if no italic code is suitable) *Italic = codes that make up the subtheme*

**Theme**	Subtheme/code	*Sub-sub theme/code*	Definition
**Comfort with demands of administrative role**	Enhanced role of admin (ERA)	*training for ERA in house*	Training organised by the practice or on-the-job training
		training for ERA ex-house	External training offered by PCN/CCG or outside provider.
		experience in administrative role	Relates to the time spent in the current role and level of familiarity with duties
		limits of admin role	Relates to what admin staff feel comfortable doing and where work strays into clinical territory.
	Protocols	*protocol implementation*	Application or failure of application of extant protocols.
		protocol disparity between actors	Dichotomy between description of protocol from different interviewees at same site.
		presence/absence of protocol	Relates to the existence of protocols.
		organisational norms (unwritten protocols)	Relates to 'its always done this way around here'. When explicitly referring to how things are done when there is not a protocol
	Role of colleagues in provision of support of knowledge		Informal support from close workmates for day-to-day activities as distinct from training.
**Environment**	Resources		Provision of physical resources such as desk/scanners/printers.
	Workspace		Relates to the physical work area in which activity is undertaken.
	Interruptions		Relates to whether staff have protected time and space for undertaking tasks
**General Practice team dynamics**	Double checking/duplication		Relates to same work being undertaken by more than one staff member.

		lack of trust	Relates to staff feelings of need to have dual operators in case someone makes a mistake.
		top of tree check	Relates to staff feelings that GPs have knowledge that is over and above other team members.
		diligence	Relates to staff personal characteristics and internal need to check/carefully process documents.
	Pastoral Support from colleagues		Relates to emotional or physical support from colleagues, as distinct from support of knowledge.
	Power and control		Relates to how managerial/GP staff decide on which work activity is suitable for which staff role and perceptions of how this is done. Distinct from protocols.
	Roles (additional roles in general practice)	Role of pharmacist in general practice	All content relating to pharmacists in general practice role, includes training. Excludes community pharmacy content
		Role of social prescriber in GP	All content relating to social prescribing in general practice role, includes training.
	Role sharing (inter-personal relationship)		Relates to how work-buddies interact with each other (as distinct from physical organisation of workload).
	Trust		Relates to how staff work together and how much they feel able to pass work safely between themselves.
**Interactions with other providers**	Interaction with secondary care	*Clarity of discharge summary*	Relates to quality/content of physical document and perceived poor quality of communication between hospital and patient.
		Shared care	Relates to care which crosses hospital/GP boundary for which there is a joint responsibility. Also includes formal ‘shared-care agreements’.
		transfer of workload	Relates to unreasonable expectation of hospital in relation to primary care follow-up care after discharge.
	Primary care services outside GP		Relates to interaction with district nurses, ambulance staff or other community provider. As distinct from interaction with carers (paid or unpaid).
**Interaction with patients**	Access (admin initiated)		Does not include receptionist as third-party conduit of information, this code is for true admin initiated contact from receptionists who know the patient well
	Access barriers		Descriptions of factors that make it harder for patients to access care.

	Access (clinician initiated)	*GP initiated*	Interactions with patients that are initiated by clinicians of different roles (includes access to do with medications).
		pharmacy initiated	
		pharmacy technician initiated	
	Access facilitators		
	Access (family/informal carer-initiated)		Interactions with the surgery that are initiated by carers.
	Access (patient initiated)	*reasons for patient-initiated access*	Interactions with the surgery that are initiated by patients. Sub code on
	Continuity of care		Relates to ongoing care provided by same clinician, one that is usually known to the patient.
		expertise of specific GPs	Relates to when patients or staff seek out particular GPs for their perceived specialist roles/interests/characteristics.
		Continuity expertise of administrators	Relates to when patients seek out particular receptionists because they know/trust them, as distinct from clinical continuity.
	Enhanced access	*criteria for enhanced access*	Relates to need for some patients to have easier access to GP services after discharge. Includes factors other than frailty e.g. language barrier.
		frailty	Relates to differences (or lack of differences) in GP care for frail patients.
		hospital practices for frailty	Relates to differences (or lack of differences) in hospital care for frail patients.
		safeguarding	Relates to perceived need to offer enhanced services/access where there is a safeguarding issue.
	Patients' health literacy		Relates to descriptions of health literacy related factors as perceived by staff. Where focus is on barriers to access use code above.
**IT in general practice**	Coding		Relates to Read or Sno-med coding activity in general.
		coding roles	Who codes what and why do they take on this role?
		coding as a subordinate activity	Relates to how coding is delegated to admin and potential negative feelings about this.

		coding impact on status	Relates to how admin staff feel they are perceived if they add coding skills to their role.
		coding for data quality	Relates to perceived importance of coding for accuracy of medical record.
		coding for patient safety	Relates to the perceived knock on effect of coding on patient safety.
		coding training	apparent
		coding updated approach	Relates to changes since Sno-med or intellisense or any software which automatically codes.
	Functionality of IT	*experience of IT*	Relates to how IT works for staff and patients.
		Intellisense role	Relates specifically to how software known as *Intellisense* works for staff and patients.
		safety features of IT	Relates to the features of IT that affect patient safety.
**Workload**	Role sharing		Relates to the functional set-up of job sharing or the benefits/disadvantages of same.
		Cover	Relates to subset of role sharing that is purely temporary. Cover of role when staff member absent.
	Segmentation		Relates to how staff split up their workload including when they do certain activities, including medicines reconciliation as a separate activity.
	Staff resource		Relates to numbers of staff or quality of staff or time resource of staff from a managerial perspective.
		recruitment difficulties	Relates to subset of staff resource which is about recruitment problems.
		GP scarce resource	Relates to particular problems with GP staff resource and need to protect GPs from work which can be done by others.
		Pharmacist resource	Relates to pharmacist staff resource.
	Time pressure		Relates to feeling of time pressure in relation to activities described.
		importance of avoiding duplication	Relates to avoiding different actors doing the same work due to time pressures or lacking staff resource.

		ability to cope with workload	Relates to ability to complete work given in time allotted. Does not include training issues. Includes GPs overlooking things due to time pressure.
	Variability		Relates to how workload is different at different times or the unpredictability of workload.

Supplementary Table 1 (S1) - Summary of practices and informal conversations

Practice Code	Date of visit	IMD deprivation decile (from PHE fingertips)	CQC rating	List size (rounded to nearest 100 for anonymity)	Role of staff member(s) observed	M/F	Age	Ethnicity	Length of time in current role	Duration of observation	FRAM model type
10	26/07/22	5th more	Good	10,700	Workflow lead (lead administrator)	F	60	White British	2 ½ years	45 minutes	Pharmacist Led – Type B
					GP	M	36	White British	8 years	35 minutes	
11	27/07/22	5th more	Outstanding	19,900	Service delivery manager	F	27	White British	6 months (but 6 years at another GP surgery in similar role)	1 hour	Combination of GP Led /Administrative Led – Type A/C
					Docman clerk	F	20	White British	7-8 months	30 minutes	
					GP partner	M	46	British Indian	17 years	20 minutes	
12	03/08/22	5th less	Good	11,800	Coding clerk	F	59	White British	4 years	1 hour (combined with pharmacy technician)	Combination of Pharmacist Led /Administrative led - Type B/C
					Pharmacy technician	F	34	White British	4 months	1 hour (combined with coding clerk)	
					Lead GP	M	45	Arab	14 years	30 minutes	

13	13/09/22	4th more	Good	24,400	Administrator responsible for *Docman* workflow	F	62	White British	3 years	45 minutes	Combination of GP led/ Pharmacist Led -Type A/B
					Administrator responsible for *Docman* workflow	F	58	White British	Approx. 20 years	20 minutes	
					Clinical pharmacist	M	38	British Indian	18 months	45 minutes	
14	12/07/22	3rd more	good	8,600	Admin lead – data management	F	36	British Indian	15 years	1 hour	Administrative led – Type C
					GP partner	M	33	White British	5 years	20 minutes	
15	20/07/22	4th less	good	9,400	Admin clerk & safeguarding lead	F	37	White British	11 years	1 hour	Administrative led – Type C
					Lead GP	M	55	British Indian	25 years	10 minutes	
					Deputy manager	F	48	British Indian	20 years	40 minutes	
16	12/10/22	least	outstanding	12,000	*Docman* administrator	M	22	White British	9 months	60 minutes	Combination of Pharmacist Led /Administrative led - Type B/C
					Clinical pharmacist	M	32	British Indian	2 months at practice, 2 years in current role	30 minutes	
					GP partner	F	58	White British	30 years	15 minutes	
17	25/10/22	3rd less	good	7,500	Receptionist and administrator	F	57	White British	4 years	30 minutes	GP led – Type A

					GP partner	F	43		6 years as a GP, 4 years as a partner	20 minutes	
18	06/10/22	3rd less	good	5,500	Lead GP	M	52	British Indian	17 years	40 minutes	GP led – Type A
					Lead administrator and receptionist	F	55	White British	20 years	60 minutes	
19	14/10/22	4th less	good	11,800	GP partner	M	33	White British	3 months as partner (joined West Side 1½ years ago)	25 minutes	Pharmacist Led – Type B
					Clinical pharmacist	F	36	British Indian	18 months	45 minutes	
					Administrative staff member	F	42	White British	2½ years	55 minutes	

Supplementary Box 2 (S2) - Type A FRAM Functions and their aspects This list of functions was used to create the Type A FRAM model.

Name of Function	Prepare for receipt of discharge summary
**Description**	General Practice has systems in place to prepare for receipt of the discharge summary. This is a background function; it is stable while discharge summaries are received and does not change during discharge summary processing. The outputs from this function are key resources for subsequent functions
**Output**	Trained members of staff to process discharge summaries
	IT systems in place for receipt and transfer of discharge summary
	Protocol in place for handling summary
	Discharge summary sent to General Practice, either electronically or via post
**Name of Function**	**Receive discharge summary**
**Description**	The discharge summary is received at the General Practice, primarily electronically. This function also includes the receipt of paper summaries, which are scanned and checked for duplicates by administrative staff
**Input**	Discharge summary sent to General Practice, either electronically or via post
**Output**	Discharge summary received by patient's clinical general practice team
**Resource**	Trained members of staff to process discharge summaries
	IT systems in place for receipt and transfer of discharge summary
**Control**	Protocol in place for handling summary
**Name of Function**	**Admin staff check Intellisense template for accuracy**
**Description**	Where Intellisense is used by the practice, admin staff check through the template, checking the correct date and clinical code has been applied
**Input**	Discharge summary received by patient's clinical general practice team
**Output**	Intellisense template updated with correct date and clinical code
**Resource**	Trained members of staff to process discharge summaries
	IT systems in place for receipt and transfer of discharge summary
**Precondition**	Discharge summary sent to General Practice, either electronically or via post
**Name of Function**	**Admin staff identify actions for the GP**
**Description**	Admin staff read through and highlight any actions for the GP (in no particular colour), including any medication changes.
**Input**	Discharge summary received by patient's clinical general practice team
**Output**	Actions for GP identified
**Precondition**	Intellisense template updated with correct date and clinical code

**Name of Function**	**Send discharge summary to GP**
**Description**	The discharge summary is sent on to the GP. This is done be sending to a central inbox to which all summaries are sent, or to a specific GP dependent on the practice's protocol in place for handling the summary
**Input**	Actions for GP identified
**Output**	Discharge summary received by GP or central inbox
**Control**	Protocol in place for handling summary
**Name of Function**	**GP reads through discharge summary**
**Description**	**The GP reads through the summary to get an idea of what has happened and what needs to be done**
**Input**	Discharge summary received by GP
**Output**	GP understanding of discharge summary
	Actions identified
**Name of Function**	**GP performs SNOMED coding and updates patient record in EMIS**
**Description**	In the type A model, the administrative staff do not undertake SNOMED coding. The GP instead codes the diagnosis and updates the record in EMIS as necessary
**Input**	GP understanding of discharge summary
**Output**	Discharge summary coded
	Patient record updated with information from discharge summary
**Name of Function**	**GP performs medication reconciliation**
**Description**	The GP compares medication on the discharge summary with those on the patient medical record, and updates as necessary. New medications are started as needed, and medications are optimised as required
**Input**	Discharge summary coded
**Output**	Medication reconciliation undertaken
	Patients on new medications contacted
**Name of Function**	**Follow through actions listed on discharge summary**
**Description**	Actions listed on the summary, such as the need to organise home visits, or the need to organise blood tests are followed through and sent to the most appropriate member of the general practice team
**Input**	Actions identified
**Output**	Patients on new medications contacted
	Reception staff (including administrative staff and secretaries) tasked as necessary by GP
	Patients contacted as necessary
	Actions followed through
**Name of Function**	**Reception staff follow through tasks**

**Input**	Reception staff (including administrative staff and secretaries) tasked as necessary by GP
**Output**	All tasks completed as requested by GP
**Name of Function**	**File discharge summary**
**Description**	Once all tasks and actions have been followed through, the discharge summary is filed. This may be done by the GP or by administrative staff, depending on the workflow within the individual practice.
**Input**	All tasks completed as requested by GP
	Actions followed through
**Output**	Discharge summary filed
**Preconditions**	Medication reconciliation undertaken
	Diagnoses coded
	Patient record updated with information from discharge summary
	Patients on new medications contacted
	Patients contacted as necessary

<u>Supplementary Box 3 (S3) - Type B FRAM Functions and their aspects</u>

This list of functions was used to create the Type B FRAM model.

**Name of Function**	**Prepare for receipt of discharge summary**
**Description**	General Practice has systems in place to prepare for receipt of the discharge summary. This is a background function; it is stable while discharge summaries are received and does not change during discharge summary processing. The outputs from this function are key resources for subsequent functions
**Output**	Trained members of staff to process discharge summaries
	IT systems in place for receipt and transfer of discharge summary
	Protocol in place for handling summary
	Discharge summary sent to General Practice, either electronically or via post
**Name of Function**	**General Practice receive discharge summary**
**Description**	The discharge summary is received at the General Practice, primarily electronically. This function also includes the receipt of paper summaries, which are scanned and checked for duplicates by administrative staff
**Input**	Discharge summary sent to General Practice, either electronically or via post
**Output**	Discharge summary received by patient's clinical general practice team
**Resource**	Trained members of staff to process discharge summaries

	IT systems in place for receipt and transfer of discharge summary
**Control**	Protocol in place for handling summary
**Name of Function**	**Admin staff check Intellisense template for accuracy**
**Description**	Where Intellisense is used by the practice, admin staff check through the template, checking the correct date and clinical code has been applied
**Input**	Discharge summary received by patient's clinical general practice team
**Output**	Intellisense template updated with correct date and clinical code
**Resource**	Trained members of staff to process discharge summaries
	IT systems in place for receipt and transfer of discharge summary
**Precondition**	Discharge summary sent to General Practice, either electronically or via post
**Name of Function**	**Admin staff code the discharge summary and record information from the summary to patient facing record**
**Description**	Key information from the discharge summary is recorded on the patient's record, such as diagnoses and test results.
**Input**	Discharge summary received by patient's clinical general practice team
**Output**	Patient record updated with some key information from discharge summary
**Precondition**	Intellisense template updated with correct date and clinical code
**Name of Function**	**Admin staff identify medication changes**
**Description**	Admin staff read through the summary and cross check medications from the discharge summary with those in EMIS
**Input**	Discharge summary received by patient's clinical general practice team
**Output**	Medication changes identified
**Precondition**	Patient record updated with some key information from discharge summary
**Name of Function**	**Send discharge summary to pharmacist**
**Description**	The discharge summary is sent on to the clinical pharmacist. All summaries with new medications or medication changes are sent to the pharmacist
**Input**	Medication changes identified
**Output**	Discharge summary received by pharmacy team
**Control**	Protocol in place for handling summary
**Name of Function**	**Pharmacist reads through discharge summary**
**Description**	The pharmacist reads through the summary to get an idea of what has happened and what needs to be done
**Input**	Discharge summary received by pharmacy team
**Output**	Pharmacist understanding of discharge summary
	Medication changes identified
	Complex summaries identified

	Need for onward referrals identified
**Name of Function**	**Pharmacist performs medication reconciliation**
**Description**	The pharmacist compares medication on the discharge summary with those on the patient medical record, and updates as necessary. New medications are started as needed and are linked to the appropriate problem on the patient record. A clinical check is performed on all medications to ensure they are all appropriate.
**Input**	Medication changes identified
**Output**	Medication reconciliation undertaken
	Summaries with new medications identified
	Summaries with multiple complex medications identified
**Name of Function**	**Pharmacist contacts patients who have had medication changes**
**Description**	Patients are contacted either via text message or phone call depending on the individual patient.
**Input**	Medication changes identified
**Output**	Patients contacted as necessary
**Precondition**	Pharmacist understanding of discharge summary
**Name of Function**	**GP processes discharge summary**
**Description**	Summaries with new medications, multiple complex medications, worsening progression of disease, cancer diagnosis or with a need for onward referrals are sent to the GP from the pharmacist
**Input**	Summaries with new medications identified
	Summaries with multiple complex medications identified
	Complex summaries identified
	Need for onward referrals identified
**Output**	New medications issued
	Onward referrals made as necessary
	Reception staff tasked as necessary
**Name of Function**	**Reception staff follow through tasks**
**Input**	Reception staff tasked as necessary
**Output**	All tasks completed as requested
**Name of Function**	**File discharge summary**
**Description**	Once all tasks and actions have been followed through, the discharge summary is filed. This may be done by the GP or by administrative staff, depending on the workflow within the individual practice.
**Input**	All tasks completed as requested
**Output**	Discharge summary filed

**Preconditions**	New medications issued
	Onward referrals made as necessary
	Patients on new medications contacted
	Patients contacted as necessary

Supplementary Box 4 (S4) - Type C FRAM Functions and their aspects

This list of functions was used to create the Type C FRAM model.

**Name of Function**	**Prepare for receipt of discharge summary**
**Description**	General Practice has systems in place to prepare for receipt of the discharge summary. This is a background function; it is stable while discharge summaries are received and does not change during discharge summary processing. The outputs from this function are key resources for subsequent functions
**Output**	Trained members of staff to process discharge summaries
	IT systems in place for receipt and transfer of discharge summary
	Protocol in place for handling summary
	Discharge summary sent to General Practice, either electronically or via post
**Name of Function**	**Receive discharge summary**
**Description**	The discharge summary is received at the General Practice, primarily electronically. This function also includes the receipt of paper summaries, which are scanned and checked for duplicates by administrative staff
**Input**	Discharge summary sent to General Practice, either electronically or via post
**Output**	Discharge summary received by patient’s clinical general practice team
**Resource**	Trained members of staff to process discharge summaries
	IT systems in place for receipt and transfer of discharge summary
**Control**	Protocol in place for handling summary
**Name of Function**	**Admin staff check Intellisense template for accuracy**
**Description**	Where Intellisense is used by the practice, admin staff check through the template, checking the correct date and clinical code has been applied
**Input**	Discharge summary received by patient’s clinical general practice team
**Output**	Intellisense template updated with correct date and clinical code
**Resource**	Trained members of staff to process discharge summaries
	IT systems in place for receipt and transfer of discharge summary

**Precondition**	Discharge summary sent to General Practice, either electronically or via post
**Name of Function**	**Admin staff add consultation to patient record**
**Description**	Administrative staff add a consultation to the patient record in EMIS to record details from the discharge summary
**Input**	Intellisense template updated with correct date and clinical code
**Output**	Consultation added to patient record for recording DS
**Name of Function**	**Admin staff code discharge summary**
**Description**	Admin staff go through the discharge summary and code whilst going through. A colour code may sometimes be used here depending on the individual practice. The consultation is used to record details from the summary. Diagnoses, blood results, and method of referral are all coded by the admin staff member
**Input**	Discharge summary received by patient's clinical general practice team
**Output**	SNOMED coding undertaken
	Medication changes identified
	Actions for GP identified
**Precondition**	Intellisense template updated with correct date and clinical code
	Consultation added to patient record for recording DS
**Name of Function**	**Admin staff check comorbidities on PMR**
**Description**	Comorbidities on the patient's record are checked and compared with those on the discharge summary. The problem list on the patient's record is updated as necessary
**Input**	SNOMED coding undertaken
**Output**	Patient record updated as necessary
**Name of Function**	**Admin staff highlight the discharge summary with actions for the GP**
**Description**	Admin staff read through the summary and highlight any actions for the GP. These are only summaries that have specific tasks for the GP and are sent to the GP only when necessary
**Input**	Actions for GP identified
**Output**	Summary highlighted with actions for GP
**Precondition**	SNOMED coding undertaken
	Patient record updated as necessary
**Name of Function**	**Admin staff send discharge summary to GP**
**Description**	Admin staff identify discharge summaries with specific actions for the GP and send these through to them. Which GP the summary is sent through to depends on the individual protocol
**Input**	Summary highlighted with actions for GP
**Output**	Appropriate GP receives discharge summary

**Control**	Protocol in place for handling summary
**Name of Function**	**Admin staff send discharge summary to pharmacy team**
**Description**	The discharge summary is sent on to the pharmacy team
**Input**	Medication changes identified
**Output**	Discharge summary received by pharmacy team
**Name of Function**	**Pharmacist performs medication reconciliation**
**Description**	The pharmacist compares medication on the discharge summary with those on the patient medical record, and updates as necessary. New medications are started as needed and are linked to the appropriate problem on the patient record. A clinical check is performed on all medications to ensure they are all appropriate.
**Input**	Medication changes identified
	Discharge summary received by pharmacy team
**Output**	Medication reconciliation undertaken
**Name of Function**	**GP processes discharge summary**
**Description**	Summaries with specific actions for the GP are processed by the GP
**Input**	Appropriate GP receives discharge summary
**Output**	Actions for GP processed
	Reception staff tasked as necessary
**Name of Function**	**Reception staff follow through tasks**
**Input**	Reception staff tasked as necessary
**Output**	All tasks completed as requested
**Name of Function**	**File discharge summary**
**Description**	Once all tasks and actions have been followed through, the discharge summary is filed. This may be done by the GP or by administrative staff, depending on the workflow within the individual practice.
**Input**	All tasks completed as requested
**Output**	Discharge summary filed
**Preconditions**	New medications issued
	Onward referrals made as necessary
	Patients on new medications contacted
	Patients contacted as necessary
